# Effectiveness of symptom perception interventions among patients with heart failure: a systematic review and meta-analysis

**DOI:** 10.3389/fcvm.2026.1704096

**Published:** 2026-02-24

**Authors:** Xiangyu Wang, Guangju Wang, Yichen Liu, Liwen Xia, Yingnan Zhao, Lifen Mao, Xiaoqing Shi, Rulan Yin

**Affiliations:** 1 Department of Nursing, The First Affiliated Hospital of Soochow University, Suzhou, China; 2 School of Nursing, Medical College of Soochow University, Suzhou, China

**Keywords:** heart failure, symptom perception, intervention, meta-analysis, RCT

## Abstract

**Background:**

Although symptom perception appears promising for enhancing health-related quality of life in patients with heart failure (HF), no quantitative pooling of effect sizes has been described to summarize and test its efficacy on clinical outcomes. This systematic review and meta-analysis aimed to determine the effect of symptom perception interventions on HF patients’ symptom perception (primary outcome), self-care, HF knowledge, self-care efficacy, quality of life, rehospitalisation, emergency department visits, and mortality (secondary outcomes).

**Methods:**

We systematically searched four databases: PubMed, Embase, the Cochrane Library, and CINAHL, from inception to March 31, 2025. RCT studies exploring the effectiveness of symptom perception interventions among HF patients were included. The studies were independently screened and extracted by two reviewers. ROB2 was applied to assess risk bias. A meta-analysis was performed using STATA 17.0.

**Results:**

Eight articles involving a total of 1030 patients were included. Pooled results showed that for HF patients, symptom perception interventions failed to decrease rehospitalization, emergency department visits and mortality. However, such interventions significantly improved patients’ immediate post-intervention outcomes, including symptom perception (SMD: 0.579, 95% CI: 0.259–0.898, *P* = 0.000), self-care (SMD: 0.697, 95% CI: 0.436–0.959, *P* = 0.000), HF knowledge (SMD: 1.481, 95% CI: 0.270–2.692, *P* = 0.017), self-care efficacy (MD: 7.875, 95% CI: 1.054–14.695, *P* = 0.024), and ultimately enhanced quality of life (MD: −8.240, 95% CI: −16.088 to −0.392, *P* = 0.040).

**Conclusion:**

The review suggests that symptom perception interventions can improve HF patients’ symptom perception, self-care, HF knowledge, self-care efficacy, and quality of life, although they do not reduce rehospitalization, emergency department visits, or mortality. The findings provide a basis for optimizing symptom perception intervention plans for future researchers.

**Systematic Review Registration:**

https://www.crd.york.ac.uk/PROSPERO/view/CRD420251035486, PROSPERO CRD420251035486.

## 1 Introduction

Heart failure (HF), characterized by symptoms and/or signs resulting from structural and/or functional abnormalities of the heart, remains a significant global public health problem (1). It is estimated that the prevalence of HF would increase by 46% over the next ten years, affecting approximately 23 million individuals worldwide (2). Those patients suffer from dyspnea, fatigue, weakness, edema, depression (3–5), etc., which limit activities of daily living and impair health-related quality of life (6). Moreover, for them, 31.9% of one-year rehospitalization rate was reported, with an estimated one-year mortality of 24.1% (7).

HF arises from multifactorial causes such as coronary artery disease (CAD) (8), hypertension (9), obesity (10), and diabetes (11), which contribute to a complex pathophysiology. This complexity poses significant challenges for effective management. Furthermore, within cardiovascular disease discourse, health promotion and prevention efforts have historically focused less on HF, particularly its early detection and proactive management (12). Given this context, developing and implementing effective strategies for managing HF is of critical importance.

Existing evidence displayed that effective symptom management can reduce HF rehospitalization rate and mortality (13), of which symptom perception is the prerequisite (14). Symptom perception is defined as involving body listening, monitoring signs to detect physical sensations, and recognizing, interpreting, and labelling symptoms (14). It contains two components, “symptom monitoring” and “symptom analysis”. Symptom monitoring refers to the behavioral process of perceiving bodily sensations and monitoring symptoms and signs of HF. Symptom analysis involves the cognitive process of identifying, interpreting, and assigning significance to these symptoms. Research suggests that heightened symptom perception perhaps sensitizes patients to HF signs/symptoms, thereby triggering care-seeking behavior early (15), helping patients transition from passively following medical advice to actively pursuing health, establishing a sustainable path for health management. Hence, symptom perception is very significant for HF patients to achieve prognosis improvement.

However, it's widely recognized that poor symptom recognition is a notable characteristic of this population. On the one hand, only 14% and 9% adhered to daily weighing and symptom monitoring, respectively (6). On the other hand, due to the early subtle signs being too vague to interpret and label accurately, most HF patients had trouble recognizing an exacerbation of HF symptoms, such as loss of resiliency, loss of consciousness, faintness, and dizziness (16). Additionally, even if patients recognized a symptom exacerbation, such as sudden weight gain and edema, these symptoms were not interpreted as severe or important HF signs (3, 17). Blunted symptom awareness could contribute to increasing the number of emergency department visits, HF hospitalizations, and mortality (18). Thus, targeted strategies and interventions for improving symptom perception behaviors of HF patients are key priorities.

Nowadays, several symptom perception interventions have been applied in HF patients. Based on the two-component symptom perception concept, most studies selected one component or integrated the intervention into a larger self-care intervention, and only a limited number focused on a complete program. In symptom monitoring, participants were provided a paper-based symptom diary (15, 19–23) or used mobile health applications (24, 25) to record daily weight and HF-related symptoms. In symptom analysis, using a daily symptom graph (24), asking open-ended questions (26), or guiding a reflective interview (27) could help HF patients recognize and interpret symptoms. Incorporate both components. Santos (23) et al. instructed HF patients using paper graphs, monitored their weight and edema, and guided reflection questions to help HF patients recognize and interpret symptoms. However, this research only tested the feasibility and acceptability of the intervention plan. That is, the effectiveness of a complete symptom perception intervention containing both monitoring and analysis is currently unclear. Furthermore, based on the existing evidence, the effectiveness of symptom perception interventions is inconsistent currently. For example, Dorsch (28) et al. found that patients who engaged in daily self-tracking of HF symptoms by a digital platform experienced a notable enhancement in quality of life (42.6 vs. 55.7, *P* = 0.0078). However, the research from Stone (29), which utilized a ’stoplight’ tool to guide patients in managing daily HF-related symptoms, indicated no significant change in quality of life (32.06 vs. 34.88, *P* > 0.05). Summing up, the effectiveness of symptom perception interventions in HF patients is still ambiguous.

Consequently, to establish an evidence base for refining symptom perception intervention strategies, we conducted this systematic review and meta-analysis to assess intervention efficacy in patients with HF, including the symptom perception (primary outcome), self-care, HF knowledge, self-care efficacy, quality of life, rehospitalisation, emergency department visits, mortality (secondary outcomes).

## 2 Methods

This systematic review and meta-analysis was conducted following the Preferred Reporting Items for Systematic Reviews and Meta-Analysis (PRISMA) statement and flowchart (30). The protocol has been registered in the International Prospective Register of Systematic Reviews (PROSPERO) (identification number: CRD420251035486).

### 2.1 Data sources and searches

As of March 31st, 2025, PubMed (1946-), Embase (1974-), Cochrane Library (1992-), and CINAHL (1937-) databases were systematically searched to identify the effectiveness of symptom perception interventions among patients with HF, with four keywords: HF, symptom perception, self-care, and randomized controlled trial (RCT). The detailed search strategy was described in Supplementary Material S1. No restrictions were applied regarding language. The reference lists of the eligible articles were reviewed to identify additional potentially eligible studies.

### 2.2 Selection criteria

The inclusion and exclusion criteria followed the PICOS search strategy model.

#### 2.2.1 Inclusion criteria

Population (P): Adult patients with a confirmed diagnosis of HF.

Intervention (I): Interventions involving both symptom monitoring and symptom analysis.

Comparison (C): Usual care.

Outcome (O): Refers to patient health-related outcomes, at least one of the following: symptom perception (primary outcome), self-care, HF knowledge, self-care efficacy, quality of life, HF rehospitalization, HF emergency department visits, all-cause hospitalization, all-cause emergency department visits, or mortality (secondary outcomes).

Study design (S): RCT.

#### 2.2.2 Exclusion criteria

The exclusion criteria were: (a) the full text was unavailable, and (b) duplicate studies and/or data (when there are different studies in the same unit and the same sample, the most recent one was selected).

### 2.3 Data selection and extraction

Two reviewers (XYW and GJW) independently conducted the literature search, screened, and extracted data. Search results were imported into NoteExpress 4.0 for management and analysis. After duplicate checking, titles and abstracts of the literature were read carefully against the inclusion and exclusion criteria. Then, the full-text was screened to confirm eligible articles. The key extracted contents included: first author name, publication year, country, recruitment, NYHA grade, gender, experimental interventions, intervention format, delivery modes, intervention duration, follow-up time, and outcomes. If study information was missing, reviewers contacted authors by phone or email to obtain relevant data. In instances where discrepancies arose, discussions with the third reviewer (YCL) were employed to reach a consensus.

Outcomes were assessed at two time points: (a) subjective patient-reported outcomes (symptom perception, self-care, HF knowledge, self-care efficacy, and quality of life) were evaluated at the earliest time point following intervention; and/ or (b) clinical events (rehospitalization, emergency department visits, and mortality) were assessed over the longest available observation period in each study (either a post-intervention follow-up or the intervention period itself), which ranged from 1 to 12 months.

### 2.4 Risk of bias assessment

The Risk of Bias 2 (ROB2) tool, recommended by the Cochrane Collaboration, was applied to evaluate the risk of bias. It comprises five domains: bias related to the randomization process, deviations from intended interventions, missing outcome data, outcome measurement, and selection of the reported result. Each domain is assessed as having a high, low, or some concern risk of bias. The overall risk of bias in the RCTs is determined by synthesizing the assessments of the five domains. The study was graded as a low risk of bias when all domains present a low risk of bias; a moderate risk of bias when at least one domain raises some concerns; and a high risk of bias when multiple domains raise concerns (31).

### 2.5 Statistical analysis

STATA 17.0 was used for data analysis. In order to ensure the consistency of the effect size direction, we conducted a numerical conversion of the “European Heart Failure Self-care Behavior Scale” for measuring self-care outcomes. All subsequent analyses were based on the converted numerical values. For continuous outcomes measured on the same scale, pooled estimates were calculated as mean difference (MD) with 95% confidence intervals (CI); and standardized mean difference (SMD) with 95% CI were applied for different scales. For dichotomous outcomes, the risk ratio (RR) with 95% CI was computed. The level of heterogeneity was measured using the I^2^ statistic, with an *I*^2^ value <50% indicating no significant heterogeneity. In cases where no significant heterogeneity was observed, a fixed-effects model was used to calculate the pooled effect sizes. Otherwise, a random-effects model was used. Sensitivity analysis was used to search for sources of heterogeneity on the basis of the elimination-by-one method. Funnel plot and Egger's tests were introduced to quantitatively test whether the funnel plot was symmetrical if ≥10 studies were included (32).

## 3 Results

### 3.1 Identification of relevant studies

**Figure 1:**
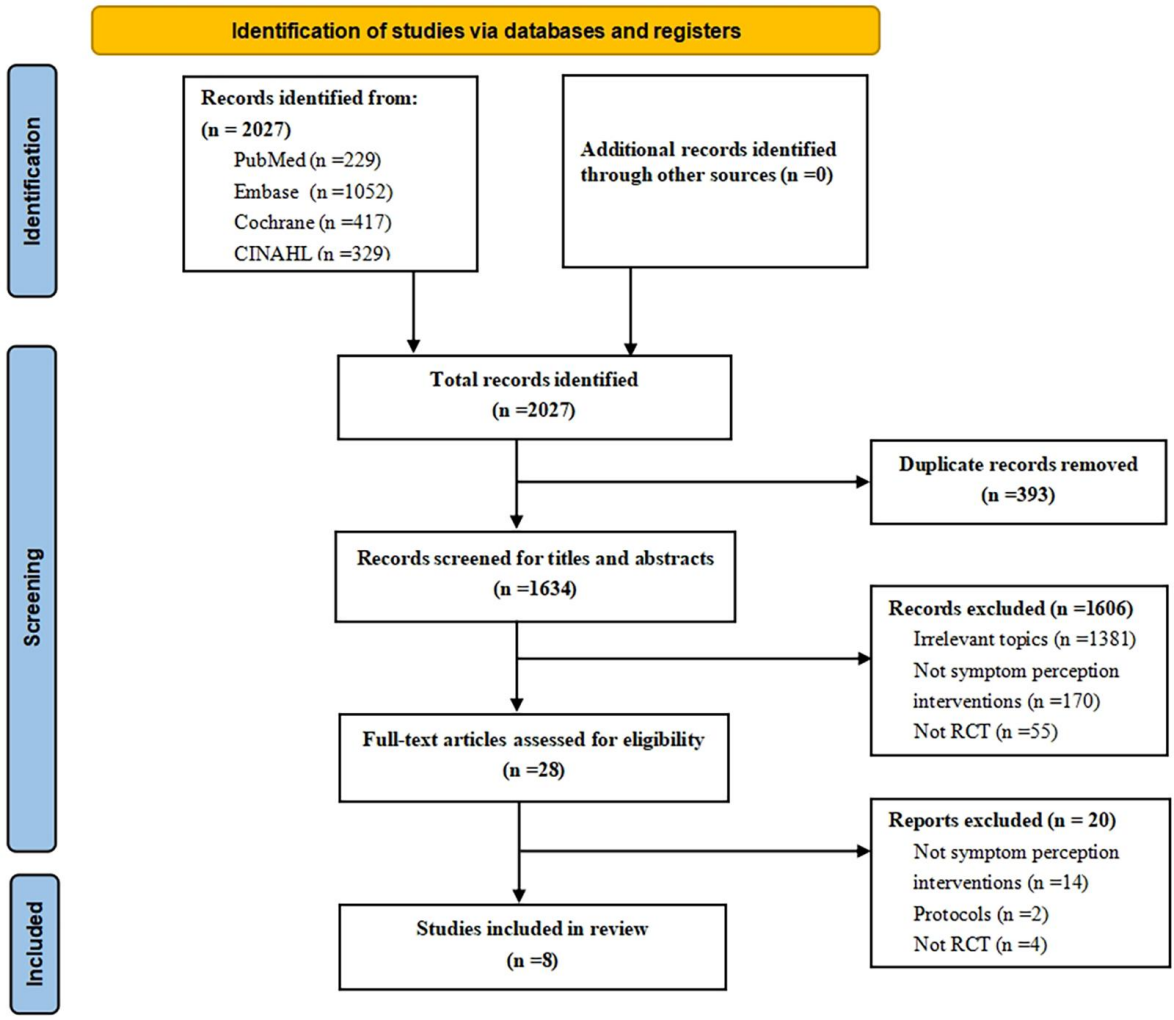
Flow diagram of study retrieval and selection progress.

A preliminary pool of 2,027 papers was confirmed from 4 databases. After the removal of 393 duplicates, the titles and abstracts of 1,634 articles were screened. At this step, 1,606 papers were excluded. As a result, 28 papers remained for further full-text analysis. Ultimately, 8 RCTs involving a total of 1,030 patients were included in this meta-analysis. The specific PRISMA flow diagram is shown in Figure 1.

### 3.2 Characteristics of included studies

A total of 8 articles (33–40) were published between 2017 and 2024, of which 6 studies (33, 34, 36, 38–40) were conducted in Western countries and 2 (35, 37) in China. The participants in each study ranged from 62 to 248. The average age of the participants ranged from 55.0 to 79.0 years of age. Regarding gender distribution, the total sample comprised 711 male participants (69.0%) and 319 female participants (31.0%). The duration of interventions ranged from 1 week (39) to 8 months (36), and the follow-up after intervention could extend up to 12 months (33). Regarding the format of interventions, seven studies conducted interventions with individuals; only one reported implementing interventions in groups (37). Furthermore, the interventions were commonly delivered through a combination of face-to-face education and telephone calls, with two exceptions: one used face-to-face education only (33), and the other utilized a tablet application (36).

The main characteristics (Table 1) and interventions (Table 2) included in the study are presented.

**Table 1:** Baseline characteristics.

Author/year	Country	Recruitment	NYHA grade	Sample size	Intervention group		Control group	
					*N*	Gender (F/M)	*N*	Gender (F/M)
Matsuda et al. (2024) (33)	Japan	Single	Ⅰ–Ⅲ	62	30	9/21	32	8/24
Nomali et al. (2024) (34)	Iran	Single	Ⅱ–Ⅳ	68	34	9/25	34	11/23
Hsu et al. (2020) (35)	China	Single	Ⅰ–Ⅲ	82	41	16/25	41	19/22
Sahlin et al. (2022) (36)	Sweden	Multicenter	Ⅰ–Ⅳ	118	58	19/39	60	28/32
Yu et al. (2022) (37)	China	Multicenter	Ⅱ–Ⅲ	236	118	39/79	118	48/70
Elpida et al. (2019) (38)	Greece	Single	Ⅰ–Ⅳ	122	61	11/50	61	9/52
Oh et al. (2023) (39)	Korea	Single	Ⅰ–Ⅳ	94	45	10/35	49	16/33
Çavuşoğlu et al. (2017) (40)	Turkey	Multicenter	Ⅱ–Ⅳ	248	125	30/95	123	37/86

**Table 2:** Symptom perception interventions and related outcomes.

Author/year	Interventions for the experimental group		Intervention formats	Delivery modes	Duration of intervention	Follow-up period	Outcomes (measurement)
	Symptom monitoring	Symptom analysis					
Matsuda et al. (2024) (33)	All participants wear wristwatch activity trackers with an accelerometer for 3 to 7 days after discharge to record activity counts and times.	Eliciting actual experiences related to HF from the patients and helping them perceive illness by having to describe it to someone else.	Individual	Face-to-face	1 month	12 months	1. Symptom perception (ESSMHF)
							1.1 Awareness and measurement (*p* = 0.942)
							1.2 Interpretation (*p* = 0.178)
							2. Self-care (EHFScBS)
							2.1 Complying with the regimen (*p* = 0.210)
							2.2 Adapting activities (*p* = 0.493)
							2.3 Asking for help (*p* = 0.716)
							3. HF rehospitalization (12 months) (7/34 vs. 9/34)^a^
							4. All-cause emergency department visits (12 months) (6/34 vs. 4/34)^a^
							5. Mortality (12 months) (0/34 vs. 1/34)^a^
Nomali et al. (2024) (34)	Using a weighing scale and a paper-based color-coded diary to monitor symptoms.	Providing education on symptom recognition related to weight changes and shortness of breath.	Individual	Face-to-face and telephone follow-up	1 month	3 months	1. Self-care (SCHFI 6.2)
							1.1 Maintenance (*p* < 0.001)
							1.2 Management (*p* < 0.001)
							1.3 Confidence (*p* < 0.001)
							2. HF knowledge (DHFKS) (*p* < 0.001)
							3. Quality of life (MLHFQ) (*p* = 0.135)
							4. All-cause hospitalization (3 months) (10/34 vs. 8/34)^a^
Hsu et al. (2020) (35)	Recording participants’ blood pressure, body weight, and symptom deterioration.	Guided participants on how to identify symptoms and helped to tailor their self-care behavior based on their records and past experiences.	Individual	Face-to-face and telephone follow-up	1 month	2 months	1. Self-care (SCHFI 6.2)
							1.1 Maintenance (*p* = 0.008)
							1.2 Management (*p* = 0.706)
							1.3 Confidence (*p* = 0.525)
Sahlin et al. (2022) (36)	Providing a tool that is based on a tablet computer, wirelessly connected to a weight scale. The participants were encouraged to use the tool to register their weight daily and assess symptoms themselves on an ordinal scale on the screen every 5 days.	Carry out interactive education based on symptom monitoring.	Individual	Tablet app	8 months	/	1. Self-care (EHFScBS) (*p* = 0.014)
							2. HF rehospitalization (8 months) (11/58 vs. 17/60)^a^
							3. HF emergency department visits (8 months) (2/58 vs. 7/60)^a^
							4. All-cause hospitalization (8 months) (21/58 vs. 29/60)^a^
							5. All-cause emergency department visits (8 months) (14/58 vs. 14/60)^a^
							6. Mortality (8 months) (5/58 vs. 5/60)^a^
Yu et al. (2022) (37)	The participants will be taught to record their daily symptom status, including body weight, peripheral oedema, and shortness of breath, on a self-monitoring form using simple methods	Teaching the participants to observe and interpret any symptom changes.	Group	Face-to-face and telephone follow-up	3 months	6 months	1. Symptom perception (SCHFI 7.2) (*p* < 0.001)
							2. Self-care (SCHFI 7.2)
							2.1 Maintenance (*p* = 0.35)
							2.2 Management (*p* < 0.001)
							2.3 Symptom perception (*p* < 0.001)
							3. HF knowledge (DHFKS) (*p* = 0.002)
							4. Self-care efficacy (SCSES) (*p* = 0.01)
							5. Quality of life (MLHFQ) (*p* = 0.001)
							6. HF rehospitalization (6 months) (17/118 vs. 24/118)^a^
							7. HF emergency department visit (6 months) (19/116 vs. 26/118)^a^
Elpida et al. (2019) (38)	Asking participants to check symptoms regularly: body weight, ankles for swelling, blood pressure, heart rate, dyspnoea, fatigue.	Based on constructivism, teaching signs and symptoms of worsening for HF patients. Such as dyspnoea, swollen ankles, weight gain, fatigue, poor concentration, loss of appetite, etc.	Individual	Face-to-face and telephone	6 months	/	1. Self-care (EHFScBS-9)
							1.1 Adhering to recommendations (*p* < 0.001)
							1.2 Fluid and sodium management (*p* < 0.001)
							1.3 Physical activity and recognition of deteriorating symptoms (*p* < 0.001)
							2. HF knowledge (AHFKT) (*p* < 0.001)
							3. Quality of life (MLHFQ) (*p* < 0.001)
							4. All-cause hospitalization (6 months) (8/61 vs. 22/61, *p* *=* 0.003)^a^
							5. Mortality (6 months) (1/61 vs. 1/61)^a^
Oh et al. (2023) (39)	Based on teach-back methods, teaching participants about weight management.	Based on teach-back methods, teaching participants about symptom management.	Individual	Face-to-face and telephone follow-up	1week	1 month	1. Symptom perception (SCHFI 7.2) (*p* < 0.001)
							2. Self-care (SCHFI 7.2)
							2.1 Maintenance (*p* = 0.001)
							2.2 Management (*p* = 0.009)
							2.3 Symptom perception (*p* < 0.001)
							3. Self-care efficacy (SCSES) (*p* = 0.689)
							4. All-cause hospitalization (1 month) (0/45 vs. 1/49)^a^
							5. All-cause emergency department visits (1 month) (0/45 vs. 1/49)^a^
Çavuşoğlu et al. (2017) (40)	Providing digital home scales with an HF education booklet for participants, telling the importance of weight monitoring and how to manage weight, and asking participants to monitor their weight.	Teaching participants how to recognize the worsening HF symptoms and when to contact the cardiologist.	Individual	Face-to-face and telephone	6 months	/	1. HF rehospitalization (6 months) (37/125 vs. 35/123, *p* = 0.95)^a^
							2. All-cause hospitalization (6 months) (47/125 vs. 43/123, *p* = 0.80)^a^
							3. All-cause emergency department visits (6 months) (26/125 vs. 40/123, *p* = 0.05)^a^
							4. Mortality (6 months) (15/125 vs. 13/125, *p* = 0.75)^a^

ESSMHF, evaluation scale for self-monitoring by patients with heart failure; EHFScBS, the European heart failure self-care behavior scale; MLHFQ, Minnesota living with heart failure questionnaire; SCHFI 6.2, Self-Care of Heart Failure Index Version 6.2; DHFKS, Dutch Heart Failure Knowledge Scale; SCHFI 7.2, 29-item Self-Care Heart Failure Index version 7.2; SCSES, The Self-care Self-Efficacy Scale; EHFScB-9, European Heart Failure Self-care Behavior Scale; AHFKT, Atlanta Heart Failure Knowledge Test.

a Group (events/N) vs. control group (events/N).

### 3.3 Risk of bias of included studies

**Figure 2:**
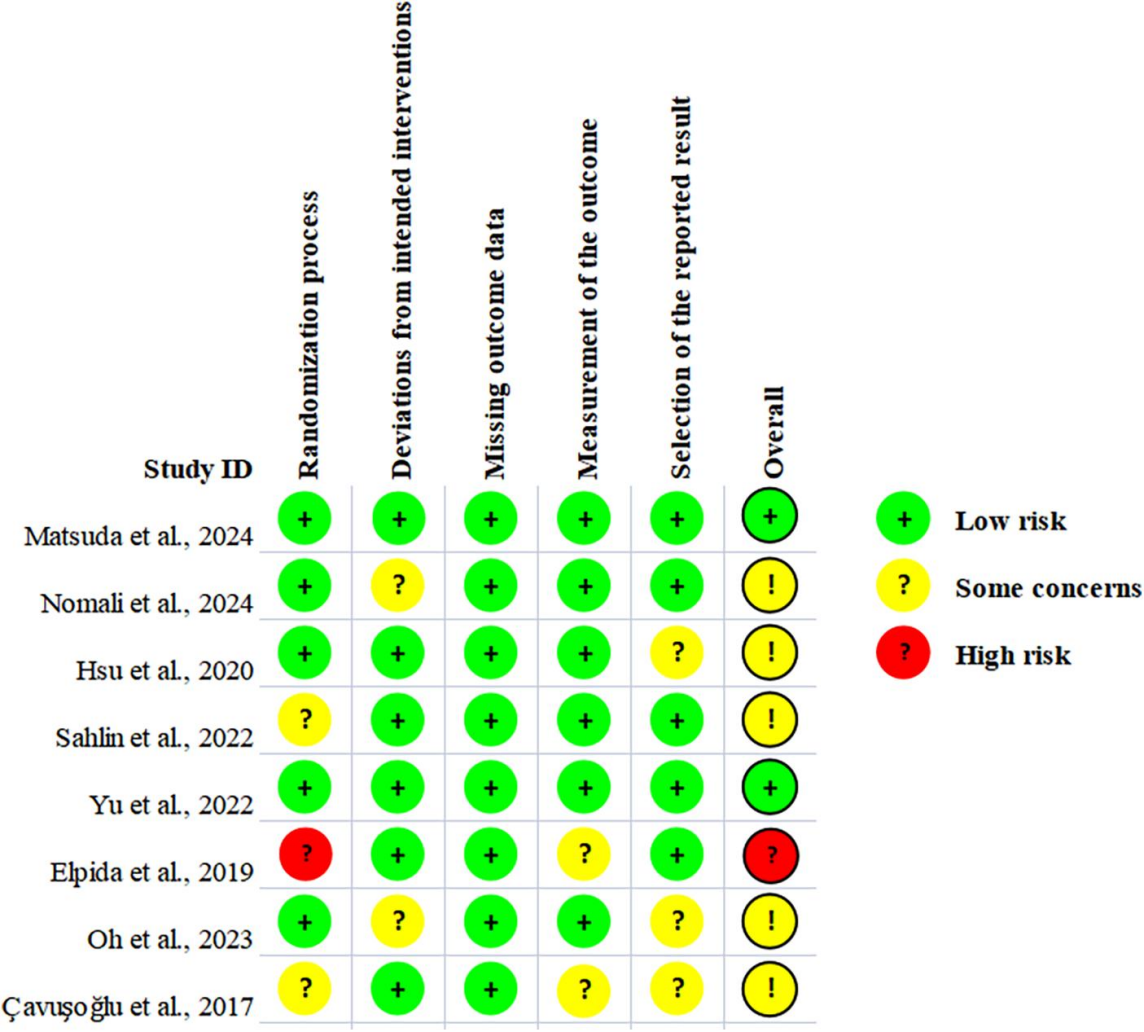
Risk of bias domains.

The results of the ROB2 assessment indicated that the majority of the included RCTs were at moderate or lower bias risk, that two of the RCTs (33, 37) were categorized as low risk, and five of the RCTs (34–36, 39, 40) were classified as having some concerns. One RCT (38) was categorized as having a high risk of bias. The primary reasons for the concerns were related to a high risk in the domains of random sequence generation and allocation concealment. The Risk of bias diagram is shown in Figure 2.

### 3.4 Effectiveness

#### 3.4.1 Primary outcome: symptom perception

**Figure 3:**
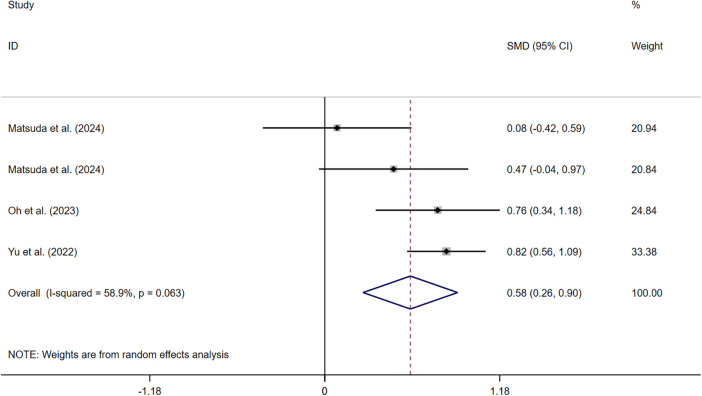
Effect of HF symptom perception.

Three studies (33, 37, 39) measured symptom perception. The pooled results indicated that symptom perception interventions led to a significant increase in symptom perception awareness (SMD: 0.579, 95% CI: 0.259–0.898, *P* *=* 0.000, *I*^2^ = 58.9%) (Figure 3). Sensitivity analysis suggested that deleting any article did not change the summary results.

#### 3.4.2 Secondary outcomes

##### 3.4.2.1 Self-care

**Figure 4:**
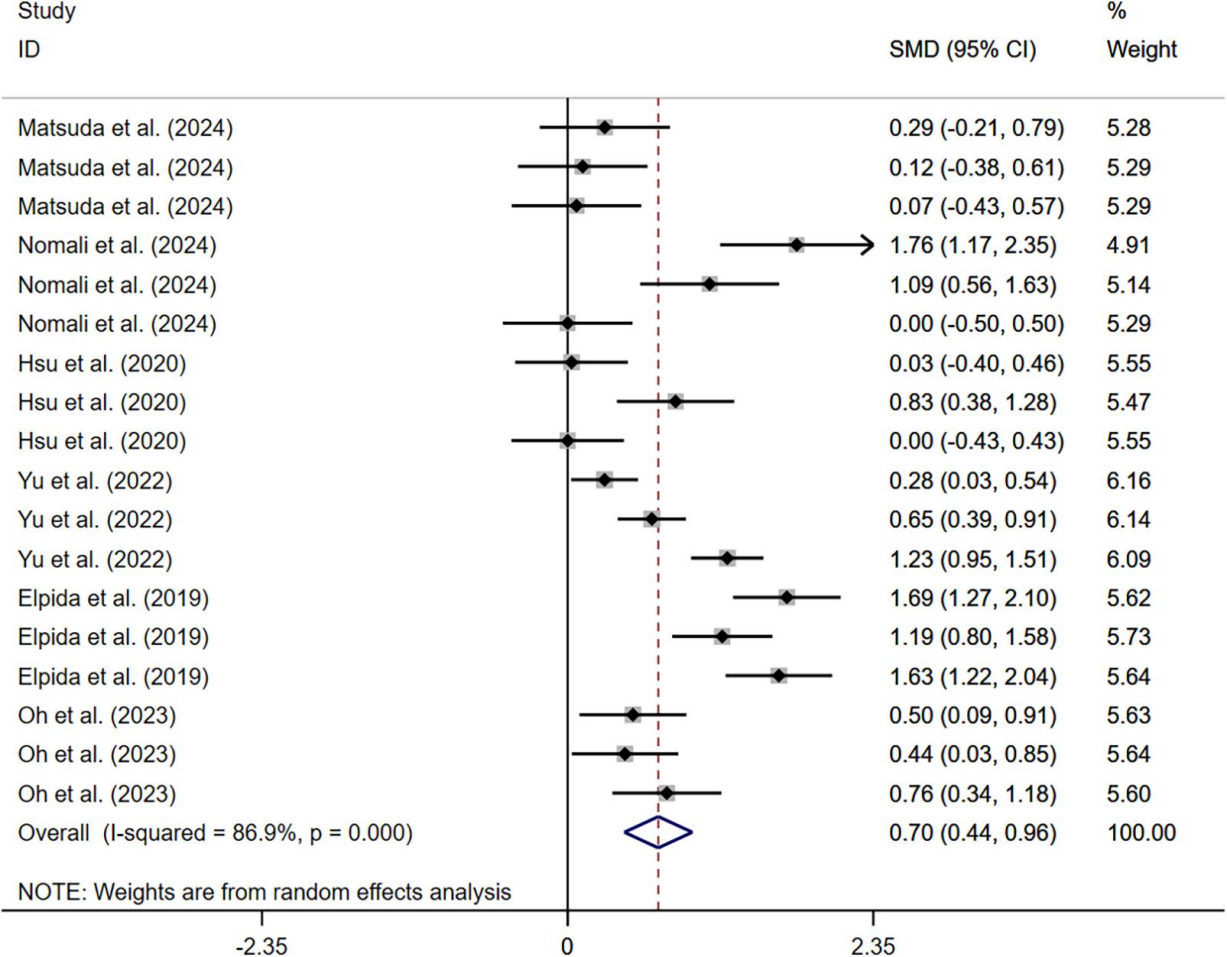
Effect of HF self-care.

Seven studies (33–39) measured self-care. One study (36) reporting first quartile and third quartile showed that symptom perception interventions led to a significant increase in self-care [IG 21.5 [13.25; 28] and CG 26 [18; 29.75], *P* = 0.014]. The pooled results of the remaining six studies confirmed this finding (SMD: 0.697, 95% CI: 0.436–0.959, *P* = 0.000, *I*^2^ = 86.9%) (Figure 4). The sensitivity analysis showed the robustness.

##### 3.4.2.2 HF knowledge

**Figure 5:**
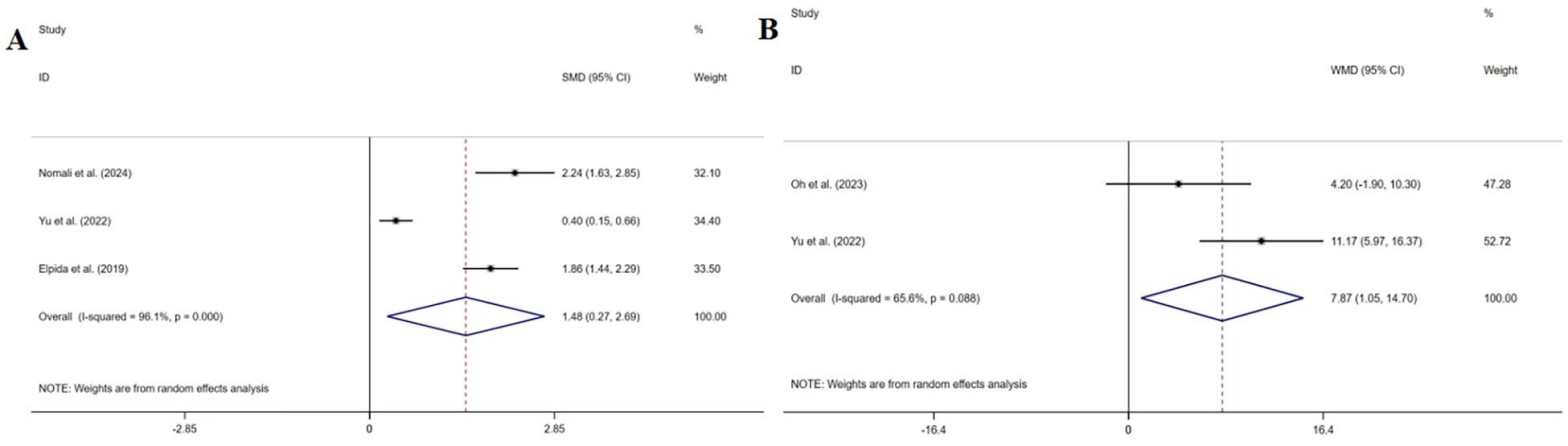
Effect of HF knowledge **(A)**, self-care efficacy **(B)**.

Three studies (34, 37, 38) measured HF knowledge. The pooled results indicated that symptom perception interventions led to a significant increase in HF knowledge (SMD: 1.481, 95% CI: 0.270–2.692, *P* = 0.017, *I^2^* = 96.1%) (Figure 5A). The sensitivity analysis showed the robustness.

##### 3.4.2.3 Self-care efficacy

Two studies (37, 39) measured self-care efficacy. The pooled results indicated that symptom perception interventions led to a significant increase in patients’ self-care efficacy (MD: 7.875, 95% CI: 1.054–14.695, *P* = 0.024, *I*^2^ = 65.6%) (Figure 5B). Sensitivity analysis suggested that deleting any article did not change the summary results.

##### 3.4.2.4 Quality of life

**Figure 6:**
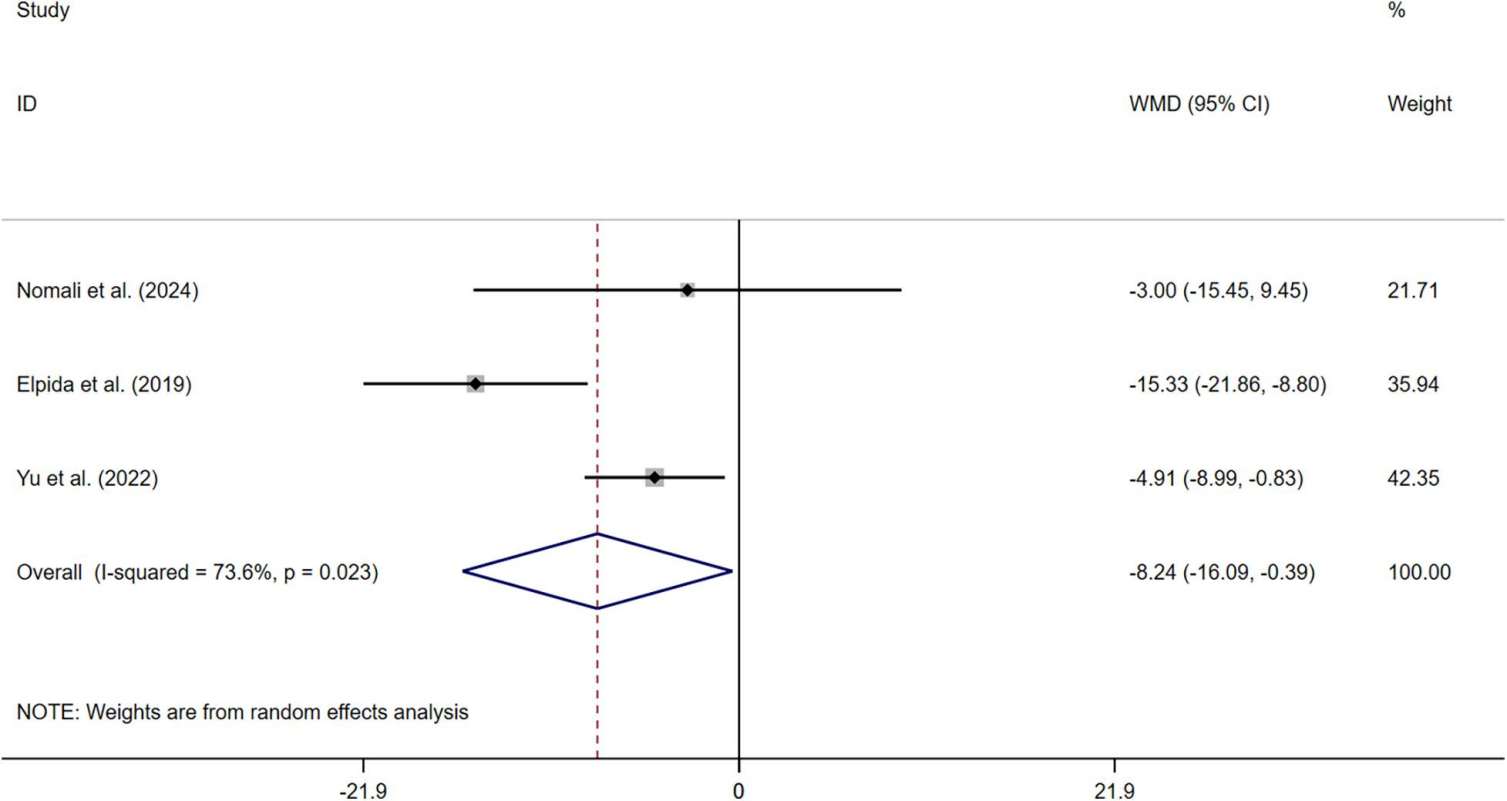
Effect of symptom perception interventions on quality of life.

Three studies (34, 37, 38) measured the quality of life. Symptom perception interventions led to a significant increase in quality of life (MD: −8.240, 95% CI: −16.088 to −0.392, *P* = 0.040, *I*^2^ = 73.6%) (Figure 6). Sensitivity analysis suggested that deleting any article did not change the summary results.

##### 3.4.2.5 HF rehospitalization

**Figure 7:**
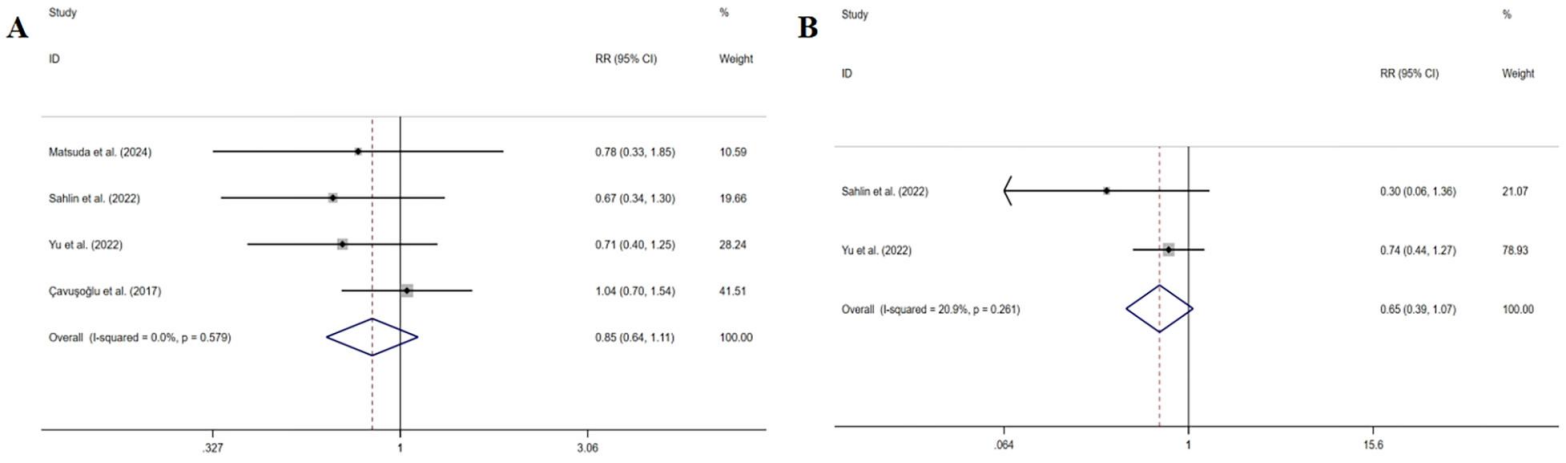
Effect of symptom perception interventions on HF rehospitalization **(A)** and HF emergency department visits **(B)**.

Four studies (33, 36, 37, 40) measured HF rehospitalization. The pooled results indicated that symptom perception interventions did not have a significant decrease in HF rehospitalization (RR: 0.846, 95% CI: 0.643–1.112, *P* = 0.230, *I^2^* = 0%) (Figure 7A). The sensitivity analysis showed the robustness.

##### 3.4.2.6 HF emergency department visits

Two studies (36, 37) measured HF emergency department visits. The pooled results indicated that symptom perception interventions did not lead to a significant decrease in HF emergency department visits (RR: 0.649, 95% CI: 0.394–1.070, *P* = 0.090, *I*^2^ = 20.9%) (Figure 7B). The sensitivity analysis indicated non-robustness. After excluding either of the two articles, the results indicated that symptom perception interventions have not led to a significant decrease in HF emergency department visits as well.

##### 3.4.2.7 All-cause hospitalization

**Figure 8:**
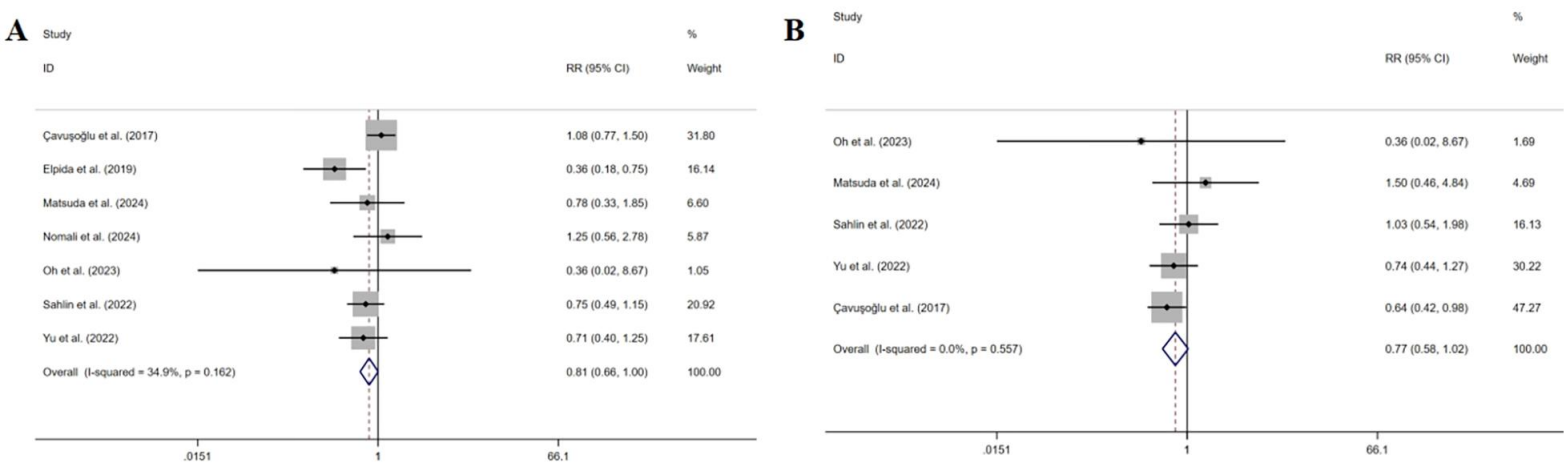
Effect of symptom perception interventions on all-cause hospitalization **(A)** and all-cause emergency department visits **(B)**.

Seven studies (33, 34, 36–40) measured all-cause hospitalization. The pooled results indicated that symptom perception interventions did not have a significant decrease in all-cause hospitalization (RR: 0.811, 95% CI: 0.657–1.000, *P* = 0.050, *I^2^* = 34.9%) (Figure 8A). The sensitivity analysis showed the robustness.

##### 3.4.2.8 All-cause emergency department visits

Five studies (33, 36, 37, 39, 40) measured all-cause emergency department visits. The pooled results indicated that symptom perception interventions did not lead to a significant decrease in all-cause emergency department visits (RR: 0.770, 95% CI: 0.580–1.023, *P* = 0.072, *I*^2^ = 0%) (Figure 8B). Sensitivity analysis suggested that deleting any article did not change the summary results.

##### 3.4.2.9 Mortality

**Figure 9:**
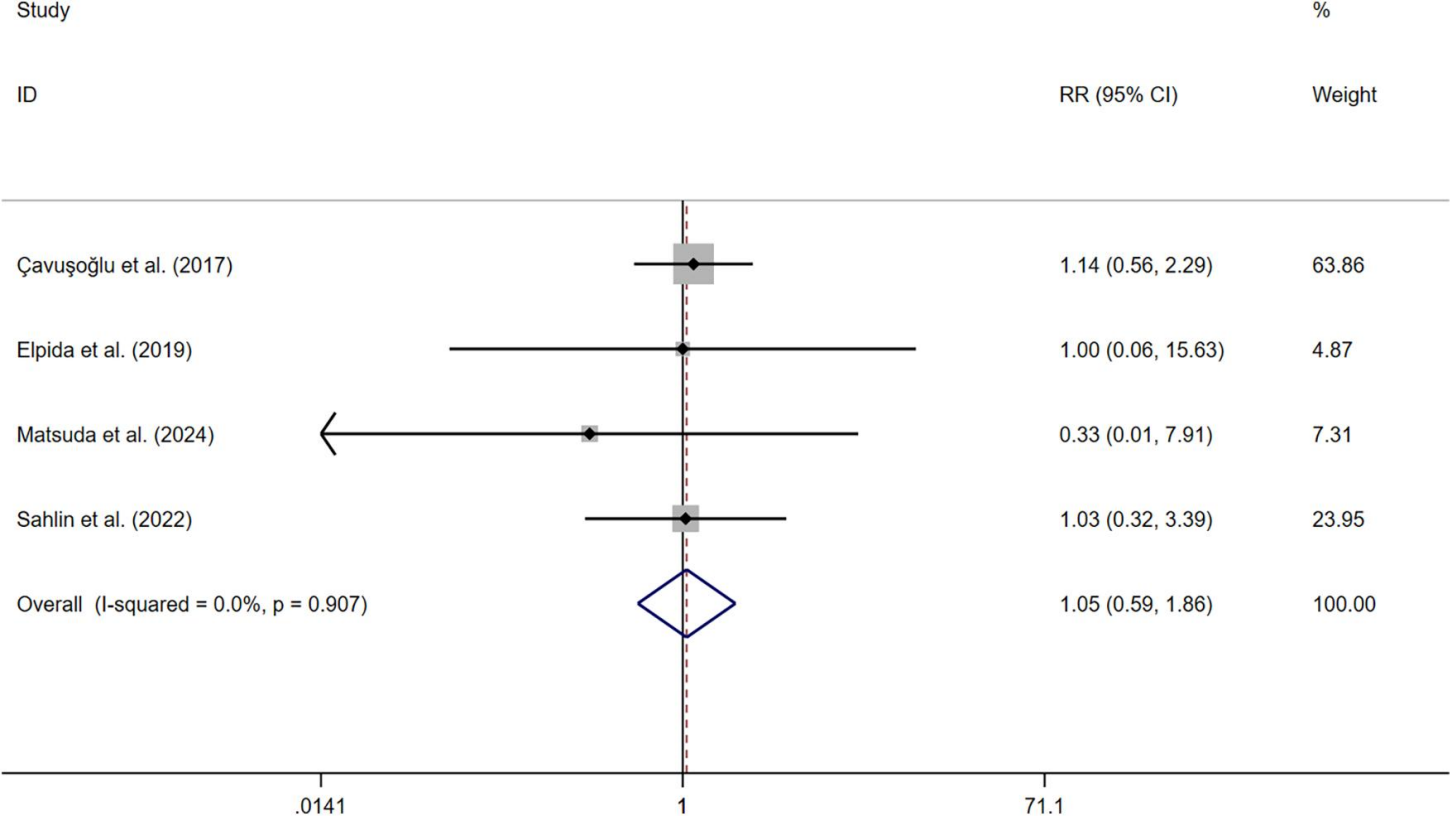
Effect of symptom perception interventions on mortality.

Four studies (33, 36, 38, 40) measured mortality. The pooled results indicated that symptom perception interventions did not have a significant decrease in mortality (RR: 1.046, 95% CI: 0.588–1.859, *P* *=* 0.878, *I*^2^ = 0%) (Figure 9). The sensitivity analysis showed the robustness. All the sensitivity analysis results can be found in Supplementary Material S2.

A summary of the effectiveness of symptom perception interventions was displayed in Table 3.

**Table 3:** The effectiveness of symptom perception interventions summary.

Outcomes	Outcomes	MD/SMD/RR	95%CI	*P*
Primary outcome	Symptom perception	0.579	0.259 to 0.898	0.000
Secondary outcomes	Self-care	0.697	0.436 to 0.959	0.000
	HF knowledge	1.481	0.270 to 2.692	0.017
	Self-care efficacy	7.875	1.054 to 14.695	0.024
	Quality of life	−8.240	−16.088 to −0.392	0.040
	HF rehospitalization	0.846	0.643 to 1.112	0.230
	HF emergency department visits	0.649	0.394 to 1.070	0.090
	All-cause hospitalization	0.811	0.657 to 1.000	0.050
	All-cause emergency department visits	0.770	0.580 to 1.023	0.072
	Mortality	1.046	0.588 to 1.859	0.878

HF, heart failure; MD, mean difference; SMD, standardized mean difference; RR, risk ratio.

## 4 Discussion

This systematic review and meta-analysis, including eight studies involving 1,030 HF patients, is the first to quantify the effect of symptom perception interventions on HF patients’ health-related outcomes. Symptom perception interventions were effective in improving symptom perception, self-care, HF knowledge, self-care efficacy, and quality of life among HF patients, while they failed to demonstrate significant improvements in rehospitalization, emergency department visits, and mortality in this population.

We found that symptom perception interventions based on two components-symptom monitoring and symptom analysis- can enhance symptom awareness in HF patients. Through symptom monitoring, such as weight monitoring (36, 40) and symptom diaries (34), patients could enhance the sensitivity of recognizing changes in HF symptoms. Through symptom analysis, such as health education reinforcement (37), reflection teaching (39), and scenario review method (33), patients could improve symptom management knowledge. Combining two components for intervention, patients’ symptom recognition capacity can be strengthened, and they have confidence in identifying and handling HF symptoms deterioration, which improves self-efficacy and symptom management ability. Higher self-efficacy is associated with better self-care (41, 42) and ultimately improved quality of life in HF patients. In light of this evidence, symptom perception interventions deserve the attention and efforts of clinical researchers. Healthcare professionals should prioritize symptom recognition, assessment, and timely response capabilities to enhance patients’ symptom perception capacity.

Our study found that symptom perception interventions had not markedly reduced rehospitalization and emergency department visits, both in HF-related and all-cause. The rehospitalization and emergency department visits among HF patients are mainly driven by the disease severity and progression (43, 44), rather than the delay in symptom recognition. That is, symptom perception has a limited impact on the disease progression. In addition, patients with HF might perceive an increase in symptoms and be able to clearly recognize that this is a sign of HF worsening (15). They understood it was time to seek help from doctors or nurses, but failed to take action due to certain reasons (45). Some patients might choose to take medication on their own instead of seeking medical treatment because they are afraid of becoming a burden on their family, or they need to take care of family and loved ones (46). Others might forgo or delay seeking medical care due to financial constraints or reluctance to impose on healthcare providers, contributing to avoidable rehospitalization and emergency department visits (47). Those actions related to delayed care-seeking led to no improvement to patient's clinical outcome, even increasing the risk of rehospitalization (48, 49). Besides, this meta-analysis showed no significant effects of interventions on decreasing mortality. The possible reason is that symptom perception interventions mainly focus on symptom monitoring and the identification and interpretation of symptoms, rather than directly treating the causes. Additionally, HF patients often have other chronic diseases (50), which accelerate the progression of the disease. Thus, other efforts should be made to reduce HF patients’ rehospitalization, emergency visits, and finally prolong their survival.

The pooled results showed no impact of symptom perception interventions on HF emergency department visits (RR: 0.649, *P* = 0.090, *I*^2^ = 20.9%), and this finding was unstable by sensitivity analysis. Further analysis found that the observed heterogeneity was primarily attributable to the substantial disparity in sample sizes (118 vs. 234) between the two studies. Individually, neither study demonstrated a statistically significant effect on heart failure-related emergency visits. That is, the current evidence supports the finding that symptom perception interventions could not reduce HF emergency department visits.

This systematic review and meta-analysis offers significant advantages. By extracting symptom perception interventions for HF patients as a distinct module and integrating data from multiple countries, the study provides a broadly representative perspective, laying a solid foundation for future intervention directions.

We have to acknowledge that several limitations lie in the review. First, the concept of symptom perception was formally introduced as a novel component only after the revision of the Situation-Specific Theory of Heart Failure Self-Care. Before the theory was refined, more symptom perception interventions were integrated into the self-care interventions, which limited our ability to accurately evaluate the clinical effectiveness of symptom perception interventions in improving patient prognosis. Second, the absence of a gold standard for assessing symptom perception has led to widespread heterogeneity in measurement tools, with studies adopting various scales to evaluate symptom perception and self-care. We attempted to conduct data transformation and then perform a combined analysis. However, the heterogeneity remained very high. It is recommended that future researchers use unified assessment tools for evaluation as much as possible.

## 5 Conclusion

Although symptom perception intervention failed to decrease rehospitalization, emergency department visit, and mortality among HF patients, it can enhance the patients’ immediate post-intervention outcomes, including symptom perception, self-care, HF knowledge, self-care efficacy, and quality of life. Included studies in the review, both the intervention and follow-up period spanned considerable time frames. It is suggested that future researchers conduct more separate interventions targeting symptom perception to further verify its effectiveness on patients’ clinical outcomes and explore the optimal timing for interventions and follow-up.

## Data Availability

The original contributions presented in the study are included in the article/Supplementary Material, further inquiries can be directed to the corresponding authors.
