# Supplementary material for: Effectiveness of symptom perception interventions among patients with heart failure: a systematic review and meta-analysis
Source: Front Cardiovasc Med. 2026 Feb 24;13:1704096. doi: 10.3389/fcvm.2026.1704096 (PMC12971966; doi:10.3389/fcvm.2026.1704096)
Supplement: Supplementary file 1 [file Datasheet1.docx]

**Search strategy**

**EMBASE (1052)**

(('heart failure'/de OR 'acute heart failure'/de OR 'cardiopulmonary insufficiency'/de OR 'cardiorenal syndrome'/de OR 'congestive heart failure'/exp OR 'diastolic dysfunction'/exp OR 'forward heart failure'/de OR 'heart outflow tract obstruction'/exp OR 'heart ventricle failure'/exp OR 'heart ventricle overload'/exp OR 'high output heart failure'/de OR 'systolic dysfunction'/exp OR 'heart failure':ab,ti OR 'cardiac failure':ab,ti OR 'heart decompensation':ab,ti OR 'myocardial failure':ab,ti OR hf:ti OR chf:ti) AND (('symptom'/de OR 'asthenia'/dm_et OR 'fatigue'/dm_et OR 'weakness'/dm_et OR 'edema'/dm_et OR 'ankle edema'/dm_et OR 'leg edema'/dm_et OR 'dyspnea'/dm_et OR 'body weight'/de OR 'weight change'/de OR 'weight fluctuation'/de OR 'weight gain'/de OR 'weight reduction'/de OR 'weight variation'/de OR 'delayed diagnosis'/de OR symptom:ab,ti OR symptoms:ab,ti OR signs:ab,ti OR dyspnea*:ab,ti OR dyspnoea*:ab,ti OR breathless:ab,ti OR ((breath NEAR/2 short*):ab,ti) OR orthopnea:ab,ti OR weight:ab,ti OR fatigue:ab,ti OR asthenia:ab,ti OR weakness:ab,ti OR 'lack of energy':ab,ti OR drowsy:ab,ti OR sleepy:ab,ti OR edema*:ab,ti OR oedema*:ab,ti OR swelling:ab,ti OR worsening:ab,ti OR exacerbat*:ab,ti OR ((physical NEAR/2 sensation*):ab,ti)) AND ('self evaluation'/de OR 'awareness'/de OR 'perception'/de OR ((self NEXT/2 (evaluation OR appraisal OR assessment OR diagnosis OR decision*)):ab,ti) OR ((patient* NEXT/3 (perception* OR perceive* OR interpret*)):ab,ti) OR ((patient* NEXT/1 (recogni* OR aware* OR interpret* OR apprais* OR understand*)):ab,ti)) OR (((symptom OR symptoms OR signs OR dyspnea* OR dyspnoea* OR breathless OR 'breath shortness' OR 'short of breath' OR 'shortness of breath' OR orthopnea OR weight OR fatigue OR asthenia OR weakness OR 'lack of energy' OR drowsy OR sleepy OR edema* OR oedema* OR swelling OR worsening OR exacerbat* OR 'physical sensation*') NEAR/4 (perception* OR perceive* OR recogni* OR aware* OR interpret* OR label*)):ab,ti) OR ((((symptom OR symptoms OR signs OR dyspnea* OR dyspnoea* OR breathless OR 'breath shortness' OR 'short of breath' OR 'shortness of breath' OR orthopnea OR weight OR fatigue OR asthenia OR weakness OR 'lack of energy' OR drowsy OR sleepy OR edema* OR oedema* OR swelling OR worsening OR exacerbat* OR 'physical sensation*') NEAR/3 (detect* OR assess* OR monitor* OR manage* OR apprais*)):ab,ti) AND (self:ab,ti OR 'self care'/de OR 'self monitoring'/de)) OR 'body listening':ab,ti OR 'body awareness':ab,ti OR (((symptom OR symptoms) NEXT/1 experience*):ab,ti) OR ((somatic NEXT/3 (perception* OR recognition OR awareness)):ab,ti) OR ((((help OR care OR treatment) NEAR/3 seek*):ab,ti) AND (earl*:ab,ti OR delay*:ab,ti OR decision*:ab,ti OR behavior*:ab,ti OR symptom*:ab,ti)))) AND ('randomized controlled trial' OR 'controlled clinical trial' OR 'randomized' OR 'placebo' OR 'randomly' OR 'trial' OR 'groups')

**PubMed (229)**

("heart failure"[Title/Abstract] OR "cardiac failure"[Title/Abstract] OR "heart decompensation"[Title/Abstract] OR "myocardial failure"[Title/Abstract] OR "HF"[Title] OR "CHF"[Title]) AND ((("symptom*"[Title/Abstract] OR "signs"[Title/Abstract] OR "dyspnea*"[Title/Abstract] OR "dyspnoea*"[Title/Abstract] OR "breathless"[Title/Abstract] OR "breathless"[Title/Abstract] OR "breath shortness"[Title/Abstract] OR "short of breath"[Title/Abstract] OR "shortness of breath"[Title/Abstract] OR "orthopnea"[Title/Abstract] OR "weight"[Title/Abstract] OR "fatigue"[Title/Abstract] OR "asthenia"[Title/Abstract] OR "weakness"[Title/Abstract] OR "lack of energy"[Title/Abstract] OR "drowsy"[Title/Abstract] OR "sleepy"[Title/Abstract] OR "edema*"[Title/Abstract] OR "oedema*"[Title/Abstract] OR "swelling"[Title/Abstract] OR "worsening"[Title/Abstract] OR "exacerbat*"[Title/Abstract] OR "physical sensation*"[Title/Abstract] OR "somatic"[Title/Abstract]) AND "perception*"[Title/Abstract]) OR "perceive symptom*"[Title/Abstract] OR "perceived symptom*"[Title/Abstract] OR "symptom recogni*"[Title/Abstract] OR "symptom aware*"[Title/Abstract] OR "symptoms recogni*"[Title/Abstract] OR "body listening"[Title/Abstract] OR "body awareness"[Title/Abstract] OR "symptom experience*"[Title/Abstract] OR "symptoms experience*"[Title/Abstract] OR "somatic awareness"[Title/Abstract] OR "symptom interpret*"[Title/Abstract] OR "symptoms interpret*"[Title/Abstract] OR "symptom label*"[Title/Abstract] OR "symptoms label*"[Title/Abstract] OR (("symptom detect*"[Title/Abstract] OR "symptom assess*"[Title/Abstract] OR "symptom monitor*"[Title/Abstract] OR "symptom manage*"[Title/Abstract] OR "symptom apprais*"[Title/Abstract] OR "symptoms detect*"[Title/Abstract] OR "symptoms assess*"[Title/Abstract] OR "symptoms manage*"[Title/Abstract]) AND "self"[Title/Abstract]) OR (("patient recogni*"[Title/Abstract] OR "patient aware*"[Title/Abstract] OR "patient interpret*"[Title/Abstract] OR "patient apprais*"[Title/Abstract]) AND ("symptom*"[Title/Abstract] OR "signs"[Title/Abstract] OR "dyspnea*"[Title/Abstract] OR "dyspnoea*"[Title/Abstract] OR "breathless"[Title/Abstract] OR "breathless"[Title/Abstract] OR "breath shortness"[Title/Abstract] OR "short of breath"[Title/Abstract] OR "shortness of breath"[Title/Abstract] OR "orthopnea"[Title/Abstract] OR "weight"[Title/Abstract] OR "fatigue"[Title/Abstract] OR "asthenia"[Title/Abstract] OR "weakness"[Title/Abstract] OR "lack of energy"[Title/Abstract] OR "drowsy"[Title/Abstract] OR "sleepy"[Title/Abstract] OR "edema*"[Title/Abstract] OR "oedema*"[Title/Abstract] OR "swelling"[Title/Abstract] OR "worsening"[Title/Abstract] OR "exacerbat*"[Title/Abstract] OR "physical sensation*"[Title/Abstract]))) AND ("randomized controlled trial"[Title/Abstract] OR "controlled clinical trial"[Title/Abstract] OR "randomized"[Title/Abstract] OR "placebo"[Title/Abstract] OR "randomly"[Title/Abstract] OR "trial"[Title/Abstract] OR "groups"[Title/Abstract])

**Cochrane Library (417)**

((("Heart failure" OR "Cardiac Failure" OR "Heart Decompensation" OR "Myocardial Failure"):ti,ab OR (HF OR CHF):ti) AND (((symptom OR symptoms OR signs OR dyspnea* OR dyspnoea* OR breathless OR (breath NEAR/2 short*) OR orthopnea OR weight OR fatigue OR asthenia OR weakness OR "lack of energy" OR drowsy OR sleepy OR edema* OR oedema* OR swelling OR worsening OR exacerbat* OR (physical NEAR/2 sensation*)):ti,ab) AND (((self NEAR/2 (evaluation OR appraisal OR assessment OR diagnosis OR decision*)) OR (patient* NEAR/3 (perception* OR perceive* OR interpret*)) OR (Patient* NEAR/1 (recogni* OR aware* OR interpret* OR apprais* OR understand*))):ti,ab) OR ((symptom* NEAR/4 (perception* OR perceive* OR recogni* OR aware* OR interpret*)):ti,ab) OR ((symptom* NEAR/3 (detect* OR assess* OR monitor* OR manage* OR apprais*)):ti,ab AND self:ti,ab ) OR ("Body listening":ti,ab OR "body awareness":ti,ab OR ((symptom OR symptoms) NEAR/1 experience*):ti,ab OR (somatic NEAR/3 (perception* OR recognition OR awareness)):ti,ab) OR (((help OR care OR treatment) NEAR/3 seek*) AND (earl* OR delay* OR decision* OR behavior* OR symptom*):ti,ab))) AND ((randomized controlled trial OR controlled clinical trial OR randomized OR placebo OR randomly OR trial OR groups):ti,ab,kw)

**CINAHL (329)**

(MH "Heart Failure+" OR TI ("Heart failure" OR "Cardiac Failure" OR "Heart Decompensation" OR "Myocardial Failure") OR AB ("Heart failure" OR "Cardiac Failure" OR "Heart Decompensation" OR "Myocardial Failure") OR TI (HF OR CHF)) AND (MH "Self Diagnosis" OR ((MH "Signs and Symptoms" OR MH "Heart Failure+/CO" OR MH "Asthenia/ET" OR MH "Body Weight Changes+/ET" OR MH "Edema/ET" OR MH "Fatigue/ET" OR MH "Muscle Weakness/ET" OR MH "Dyspnea/ET" OR MH "Diagnosis, Delayed" OR TI (symptom OR symptoms OR signs OR dyspnea* OR dyspnoea* OR breathless OR (breath N2 short*) OR orthopnea OR weight OR fatigue OR asthenia OR weakness OR "lack of energy" OR drowsy OR sleepy OR edema* OR oedema* OR swelling OR worsening OR exacerbat* OR (physical N2 sensation*)) OR AB (symptom OR symptoms OR signs OR dyspnea* OR dyspnoea* OR breathless OR (breath N2 short*) OR orthopnea OR weight OR fatigue OR asthenia OR weakness OR "lack of energy" OR drowsy OR sleepy OR edema* OR oedema* OR swelling OR worsening OR exacerbat* OR (physical N2 sensation*))) AND (MH "Self Assessment" OR MH "Perception" OR TI ((self N2 (evaluation OR appraisal OR assessment OR diagnosis OR decision*)) OR (patient* N3 (perception* OR perceive* OR interpret*)) OR (Patient*N1 (recogni* OR aware* OR apprais*))) OR AB ((self N2 (evaluation OR appraisal OR assessment OR diagnosis OR decision*)) OR (patient* N3 (perception* OR perceive* OR interpret*)) OR (Patient*N1 (recogni* OR aware* OR apprais*))))) OR TI ((Symptom OR symptoms OR signs OR dyspnea* OR dyspnoea* OR breathless OR (breath N2 short*) OR orthopnea OR weight OR fatigue OR asthenia OR weakness OR "lack of energy" OR drowsy OR sleepy OR edema* OR oedema* OR swelling OR worsening OR exacerbat* OR (physical N2 sensation*)) N4 (perception* OR perceive* OR recogni* OR aware* OR interpret* OR label*)) OR AB ((Symptom OR symptoms OR signs OR dyspnea* OR dyspnoea* OR breathless OR (breath N2 short*) OR orthopnea OR weight OR fatigue OR asthenia OR weakness OR "lack of energy" OR drowsy OR sleepy OR edema* OR oedema* OR swelling OR worsening OR exacerbat* OR (physical N2 sensation*)) N4 (perception* OR perceive* OR recogni* OR aware* OR interpret* OR label*)) OR ((TI ((Symptom OR symptoms OR signs OR dyspnea* OR dyspnoea* OR breathless OR (breath N2 short*) OR orthopnea OR weight OR fatigue OR asthenia OR weakness OR "lack of energy" OR drowsy OR sleepy OR edema* OR oedema* OR swelling OR worsening OR exacerbat* OR (physical N2 sensation*)) N3 (detect* OR assess* OR monitor* OR manage* OR apprais*)) OR AB ((Symptom OR symptoms OR signs OR dyspnea* OR dyspnoea* OR breathless OR (breath N2 short*) OR orthopnea OR weight OR fatigue OR asthenia OR weakness OR "lack of energy" OR drowsy OR sleepy OR edema* OR oedema* OR swelling OR worsening OR exacerbat* OR (physical N2 sensation*)) N3 (detect* OR assess* OR monitor* OR manage* OR apprais*))) AND (TI self OR AB self OR MH "Self Care")) OR TI ("Body listening" OR "body awareness" OR ((symptom OR symptoms) W1 experience*) OR (somatic W3 (perception* OR recognition OR awareness OR sensation*)) OR (((help OR care OR treatment) N3 seek*) AND (earl* OR delay* OR decision* OR behavior* OR symptom*))) OR AB ("Body listening" OR "body awareness" OR ((symptom OR symptoms) W1 experience*) OR (somatic W3 (perception* OR recognition OR awareness OR sensation*)) OR (((help OR care OR treatment) N3 seek*) AND (earl* OR delay* OR decision* OR behavior* OR symptom*)))) AND (MH "Randomized Controlled Trials" OR MH "Clinical Trials" OR TI (randomized OR placebo OR randomly OR trial OR groups) OR AB (randomized OR placebo OR randomly OR trial OR groups) OR KW (randomized OR placebo OR randomly OR trial OR groups))
