# Supplementary material for: Effectiveness of symptom perception interventions among patients with heart failure: a systematic review and meta-analysis
Source: Front Cardiovasc Med. 2026 Feb 24;13:1704096. doi: 10.3389/fcvm.2026.1704096 (PMC12971966; doi:10.3389/fcvm.2026.1704096)
Supplement: Supplementary file 2 [file Datasheet2.docx]

The results of the sensitivity analysis are as follows:

1. **Primary outcome--symptom perception**

1. **Secondary outcome**

**2.1 Self-care**

**2.2 HF knowledge**

**2.3 Self-care efficacy**

**2.4 Quality of life**

**2.5 HF re-hospitalization**

**2.6 HF emergency department visits**

**2.7 All-cause hospitalization**

**2.8 All-cause emergency department visits**

**2.9 Mortality**
